# Screening of Anti-Prion Compounds Using the Protein Misfolding Cyclic Amplification Technology

**DOI:** 10.3390/biom14091113

**Published:** 2024-09-04

**Authors:** Sandra Pritzkow, Isaac Schauer, Ananya Tupaki-Sreepurna, Rodrigo Morales, Claudio Soto

**Affiliations:** 1 Department of Neurology, Mitchell Center for Alzheimer’s Disease and Related Brain Disorders, University of Texas Health Science Center at Houston, McGovern Medical School, Houston, TX 77030, USArodrigo.moralesloyola@uth.tmc.edu (R.M.); 2 Centro Integrativo de Biologia y Quimica Aplicada (CIBQA), Universidad Bernardo O’Higgins, Santiago 8370993, Chile

**Keywords:** prions, PMCA, Creutzfeldt-Jakob disease, therapeutic compounds, prion replication

## Abstract

Prion diseases are 100% fatal infectious neurodegenerative diseases affecting the brains of humans and other mammals. The disease is caused by the formation and replication of prions, composed exclusively of the misfolded prion protein (PrP^Sc^). We invented and developed the protein misfolding cyclic amplification (PMCA) technology for in vitro prion replication, which allow us to replicate the infectious agent and it is commonly used for ultra-sensitive prion detection in biological fluids, tissues and environmental samples. In this article, we studied whether PMCA can be used to screen for chemical compounds that block prion replication. A small set of compounds previously shown to have anti-prion activity in various systems, mostly using cells infected with murine prions, was evaluated for their ability to prevent the replication of prions. Studies were conducted simultaneously with prions derived from 4 species, including human, cattle, cervid and mouse. Our results show that only one of these compounds (methylene blue) was able to completely inhibit prion replication in all species. Estimation of the IC50 for methylene blue inhibition of human prions causing variant Creutzfeldt-Jakob disease (vCJD) was 7.7 μM. Finally, we showed that PMCA can be used for structure-activity relationship studies of anti-prion compounds. Interestingly, some of the less efficient prion inhibitors altered the replication of prions in some species and not others, suggesting that PMCA is useful for studying the differential selectivity of potential drugs.

## 1. Introduction

Prion diseases (PrDs) are fatal neurodegenerative disorders (NDs) affecting humans and various mammals, including sheep, goats, mink, cervids, cattle, felines and ungulates [1]. The underlying mechanism in PrDs involves the accumulation of the pathological form of the prion protein (PrP^Sc^) leading to brain damage in the form of spongiform encephalopathy, neuronal loss, synaptic dysfunction and brain inflammation [1]. PrP^Sc^ forms by autocatalytic conversion of the host’s normal prion protein (PrP^C^) and this process can be spread infectiously between individuals [2]. Creutzfeldt-Jakob disease (CJD) is the most common PrD in humans, and it can appear in sporadic (sCJD), familial or infectious forms. Animal PrDs include bovine spongiform encephalopathy (BSE) in cattle, chronic wasting disease (CWD) in deer and elk, and scrapie in sheep. CJD is a rare disease; however, the heretical nature of the prion infectious agent, the reported transmission of the disease between cattle and humans generating variant CJD (vCJD), and the recent expansion of the number of cases and geographical location of CWD made it important to develop strategies for efficient treatment [1]. The concept that PrP^Sc^ is the only component of the infectious material and that cerebral accumulation of PrP^Sc^ leads to neurodegeneration and disease is almost universally accepted in the field [3]. The atomic resolution structures for both PrP^C^ and PrP^Sc^ are known [4] (at least for some species) and animal models fully recapitulate all characteristics of human prion diseases [5].

Despite impressive knowledge about the molecular and cellular basis of PrDs, currently, there is not any approved treatment for inhibiting prion replication in CJD or any other prion disease [6,7]. Part of the difficulty in the development of therapeutic interventions is the lack of biologically relevant screening assays to identify candidate hit molecules. Our strategy was to use the Protein Misfolding Cyclic Amplification (PMCA) technology we previously invented and developed for in vitro prion replication [8,9] to screen for compounds capable of preventing prion formation and propagation. Over the past decade, PMCA has proven to be a great tool for studying replication of infectious prions, understanding the complex prion biology, and detecting with extremely high sensitivity tiny amounts of infectious prions [10]. The PMCA technology has enabled researchers, for the first time, to cyclically amplify the folding and biochemical properties of a protein in a manner conceptually analogous to the amplification of DNA by PCR [8,9,11]. PMCA allowed the generation of infectious prions in vitro, providing the strongest proof in favor of the prion hypothesis [10,12]. The technique has also permitted detection, for the first time, of infectious prions in the blood and urine of animals and humans, offering a great possibility for early diagnosis [13,14]. Indeed, PMCA (and a variation called RT-QuIC) are now routinely used for the diagnosis of CJD worldwide. With PMCA, it has been possible to address critical issues in the prion field, including prion strains, species barriers, and de novo generation of infectious particles [10,15,16,17]. The efficiency of PMCA and the faithfulness with which it reproduces prion biology (e.g., infectivity, strain diversity, species barriers) suggest that compounds interfering with PMCA amplification may represent good hits for therapeutic development [18]. On the contrary, in vitro, misfolded PrP particles generated de novo from recombinant proteins have been shown to adopt a different structure and it is not infectious [19,20,21]. Indeed, a study evaluating the infectivity of recPrP produced in 20,000 experiments showed that recPrP^Sc^ amyloid was readily formed in a test tube but generated no infectivity [21]. The difference in our study is that we used the PMCA technology to faithfully direct the templated conversion of PrP^C^ into PrP^Sc^. Furthermore, PMCA offers the possibility to test the effect of the compounds on multiple PrP^Sc^ strains and species, which may overcome the problem identified in previous reports that compounds can inhibit replication of prions from certain strains/species, but not others [22,23,24]. The main goal of this study was to provide proof-of-concept data that PMCA can be used as a rapid and biologically relevant in vitro screening assay to identify compounds able to block prion replication. The compounds tested were selected based on previously published results suggesting they can have anti-prion activity.

In this article, we used PMCA to test a small set of molecules which have been reported to have anti-prion activities in diverse experiments, including Congo red, quinacrine, curcumin, tannic acid, methylene blue, rhodanine, chlorpromazine and minocycline. Congo red is perhaps one of the oldest reported prion inhibitors. Congo red is the sodium salt of 3,3′-([1,1′-biphenyl]-4,4′-diyl)bis(4-aminonaphthalene-1-sulfonic acid) and is often used to stain for amyloid deposits. The first report of Congo red as a prion inhibitor was published in 1992 by Caughey and Race [25] and confirmed later in various studies using diverse model systems [26,27,28,29]. Quinacrine, an acridine derivative formerly used as an antimalarial drug, was shown in cellular models of prion replication to inhibit prion formation [30,31]. However, studies using models of human prion replication did not show significant activity [32] and clinical trials with this drug in CJD did not produce beneficial results [33,34]. Curcumin, a natural compound and a major component of the spice turmeric, was shown to inhibit the in vitro formation of protease-resistance PrP [35]. Tannic acid is a large polyphenolic compound, which is a specific form of tannin. Tannic acid is found in the nutgalls formed by insects on twigs of certain oak trees. Using cellular models and RT-QuIC, tannic acid was shown to prevent PrP^Sc^ formation [36,37]. Methylthioninium chloride, usually called methylene blue (MB), is a salt used as a dye and as a medication approved for the treatment of methemoglobinemia. MB is an inhibitor of nitric oxide synthase and guanylate cyclase. Experiments using prion-infected cells showed that MB efficiently blocked prion replication [31]. It was also shown by NMR that MB binds PrP at a surface cleft of a fibrillogenic region of the protein and prevents its aggregation [38]. Rhodanine is a 5-membered heterocyclic organic compound possessing a thiazolidine core and a derivative was shown to inhibit prion-induced neuroinflammation [39] as well as be able to inhibit Tau protein aggregation [40]. Chlorpromazine is an antipsychotic drug used to treat psychiatric disorders such as schizophrenia, which was shown to have a potent anti-prion activity in prion-infected cells [31,41]. Minocycline and other tetracyclines have been shown to interact with and reverse protease-resistant prion protein and intraperitoneal injection of the drug in a hamster model of prion disease showed an 81% increased survival time [42].

## 2. Materials and Methods

***Prion-infected brain samples***. As inocula to trigger prion replication we used brain homogenates from: (i) a wild-type mouse experimentally infected with the RML prion strain; (ii) a human affected by vCJD; (iii) a cow affected by BSE; and (iv) a white-tailed deer naturally infected by CWD. Ten percent weight/volume (*w*/*v*) brain homogenates were prepared in PBS and large debris were removed by centrifugation at 810× *g* at 4 °C for 1 min. The supernatants were aliquoted, snap-frozen in liquid nitrogen, and stored at −80 °C until use.

***Preparation of PMCA substrates.*** 10% *w*/*v* brain homogenates were prepared in conversion buffer (PBS supplemented with 1% Triton X-100, 150 mM NaCl, and Complete, EDTA-free protease inhibitor). Large debris were removed by centrifugation at 810× *g* at 4 °C for 1 min. The supernatants were aliquoted, snap-frozen in liquid nitrogen, and stored at −80 °C until use. For RML prion replication, we used wild-type mouse brains. For vCJD, we used transgenic mice expressing human *PRNP* with 129M polymorphism (Tg 6815 line) kindly provided by Dr. Glenn Telling (Colorado State University). For CWD amplification, we used gene-targeted transgenic mice expressing deer *PRNP* (Tg Gt226Q), provided by Dr. Glenn Telling. For BSE prion replication, we used as substrate transgenic mice expressing bovine *PRNP* (TgBoPrP) which was also provided by Dr. Glenn Telling.

***Prion replication by PMCA and screening of prion inhibitors***. PMCA was performed as described previously [9]. Briefly, thin PCR tubes (Eppendorf, Cat. No. 951010022) were used to perform the experiments. A 220–250 mL volume of water was poured into the sonicator holder in every experiment. Each sonication cycle comprised 20 s of sonication at an amplitude of 13 and 29 min 40 s of incubation. The horn and converter of the sonicator were placed inside a 32 °C incubator. A total of 48 PMCA cycles (24 h) were performed. To trigger prion replication, different dilutions of prion-infected brain homogenate were added to the reaction including the respective PMCA substrate (see above). The final volume of the reaction was 100 μL. At the same time, potentially inhibitory compounds (Congo red, rhodanine, quinacrine, tannic acid, methylene blue, curcumin, chlorpromazine, minocycline, azure A and thionine acetate) were added at a final concentration of 100 μM. Stock solutions of the compounds were dissolved in DMSO at 10 mM, and diluted into the reaction to reach a 1% DMSO concentration in the tube.

***Proteinase K (PK) digestion and western blotting.*** PMCA products and standard prion-laden brain homogenates were incubated with PK (100 µg/mL) for 1 h at 37 °C with agitation, using the conditions previously described in detail [9]. PK digestions were stopped by the addition of loading sample buffer and boiling for 10 min at 100 °C. Proteins were separated by SDS-PAGE and then transferred to 0.45 µm nitrocellulose membranes, which were blocked with 10% *w*/*v* dry milk for 1 h at RT and then probed with monoclonal antibody 6D11 unless stated otherwise.

## 3. Results

### 3.1. Screening of a Small Selection of Anti-Prion Compounds

**Figure 1:**
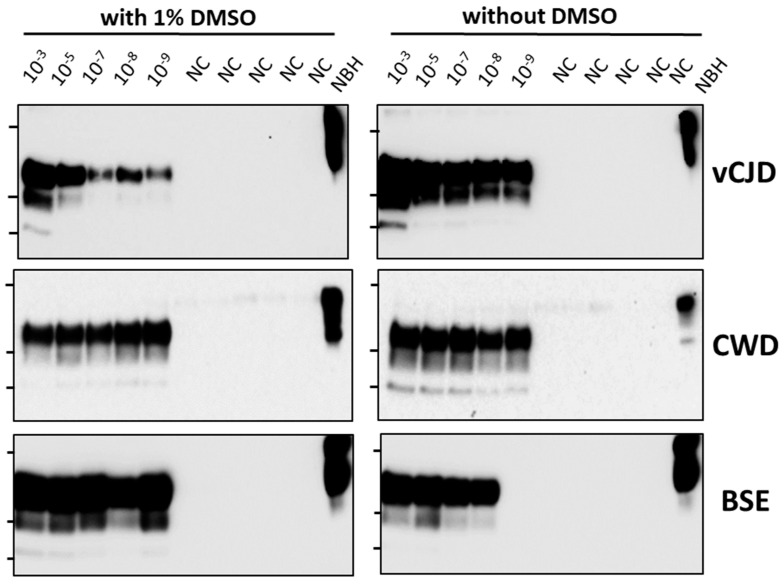
**Effect of DMSO on the efficiency of prion replication**. Since molecules to be tested are generally dissolved in DMSO, we tested whether addition of 1% DMSO (final concentration in the tube) produced any effect on prion replication. For this purpose, we incubated a series of dilutions of brain homogenate infected with vCJD, CWD and BSE with 1% DMSO and proceed to perform PMCA amplification in the presence of the respective substrate. After one round of 48 PMCA cycles (24 h), samples were analyzed by western blot after proteinase K digestion. Negative control (NC) consists of samples containing all materials except for PrPSc seeds. NBH correspond to the normal brain homogenate used for each amplification (i.e., wild type brain for rodent prions, transgenic mice expressing human PrP for vCJD prions, etc.). This is used as a migration control. Lanes on the left of each blot represent molecular weight standards (34 KDa, 26 KDa and 17 KDa). Please see the original Western blot image in the Supplementary Materials.

To assess whether PMCA can be used for identifying compounds able to prevent prion replication, we tested the activity of eight compounds previously reported to inhibit prion propagation in diverse systems, mostly cells [7]. The compounds tested were rhodanine, Congo red, quinacrine, tannic acid, methylene blue, curcumin, chlorpromazine and minocycline. All compounds were dissolved in DMSO and diluted to reach a 1% *v*/*v* concentration of DMSO in the reaction. Before testing the compounds, we first studied whether the presence of 1% DMSO might interfere with PMCA efficiency. For this purpose, serial dilutions of vCJD, CWD and BSE were tested by PMCA in the presence or absence of 1% DMSO (Figure 1). The results showed that regardless of whether the reaction was conducted with or without DMSO, PMCA was successful in amplifying up to a 10^−9^ dilution of each brain-infected material, indicating that 1% DMSO does not interfere with PMCA.

**Figure 2:**
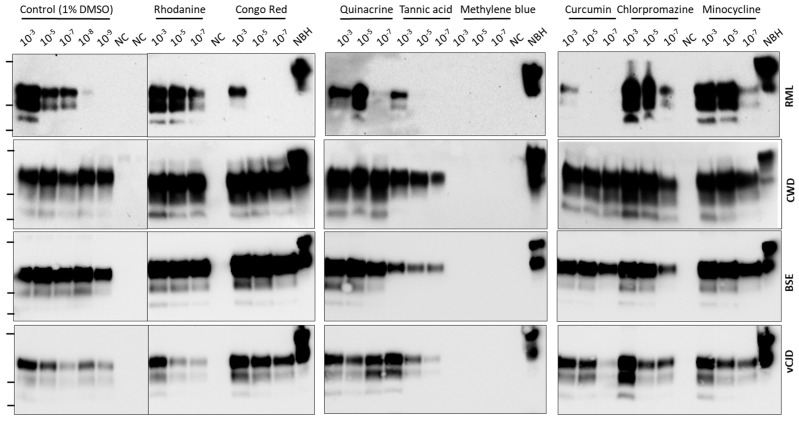
**Using PMCA to evaluate anti-prion activity.** Eight molecules previously reported as able to prevent prion replication in various models were tested at 100 μM concentration for their activity to prevent PrP^C^ to PrP^Sc^ conversion by PMCA of RML, CWD, BSE and vCJD prions. For testing inhibition we added 3 different quantities of PrP^Sc^ equivalent to a 10^−3^, 10^−5^ and 10^−7^ dilution of infected brain homogenate. Control consists on PMCA in the absence of any compound but the vehicle used to solubilize the molecules (1% DMSO). Negative control (NC) consists of samples without addition of PrP^Sc^. Samples were subjected to one round of 48 PMCA cycles (24 h) and analyzed by western blot after proteinase K digestion. NBH correspond to transgenic mice normal brain homogenate, used as a migration control. Lanes on the left of each blot represent molecular weight standards (34 KDa, 26 KDa and 17 KDa). Please see the original Western blot image in the Supplementary Materials.

Each compound was tested at a concentration of 100 μM using one round of PMCA and utilizing prions from 4 different species, including mouse RML, white-tailed deer CWD, cattle classical BSE, and human vCJD. Different dilutions of infected brain homogenate (from 10^−3^ to 10^−7^) were used to seed prion replication. As positive controls, we used the same dilutions in the absence of any compound but included 1% DMSO (the vehicle used to dissolve the compounds), which did not change in any way prion replication in any of the species studied (Figure 1 and Figure 2, left panels). The results show that only methylene blue (MB) was able to completely block prion replication in all species at this concentration (Figure 2). Interestingly, some molecules were able to inhibit prion replication in some species, but not others. For example, Congo red, tannic acid, quinacrine and curcumin partially inhibited mouse RML prion replication, but did not have any detectable effect with non-experimental prions in relevant species (Figure 2). The ability to study anti-prion compounds in various species at the same time represents one of the great advantages of using PMCA for screening.

### 3.2. Estimation of IC50 for Compounds’ Activity

**Figure 3:**
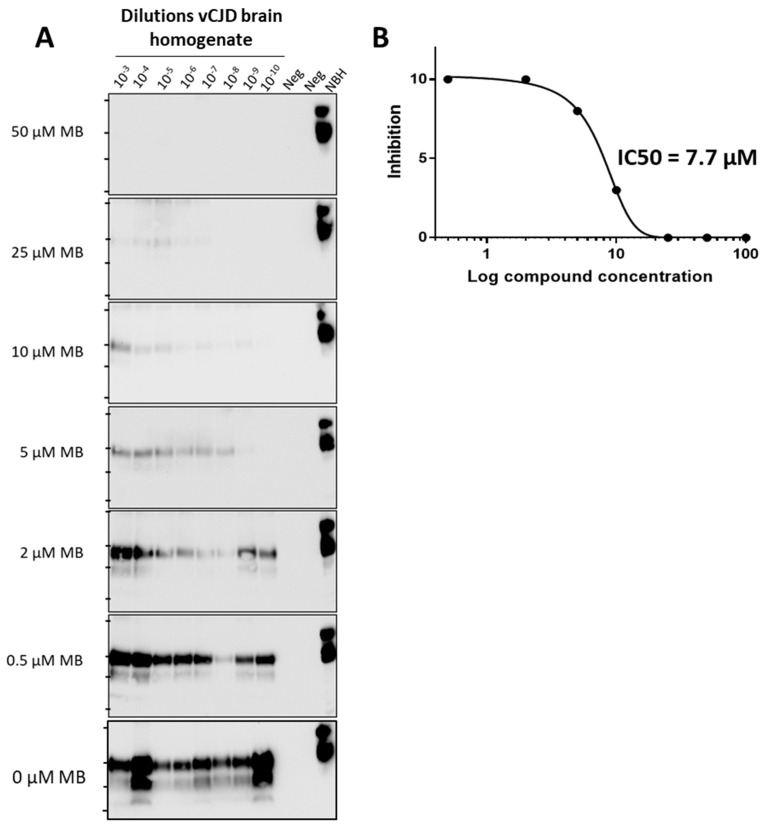
**Estimation of IC50 for methylene blue anti-prion activity**. (**A**), The effect of different concentrations of methylene blue (MB) on vCJD prion replication was evaluated at different dilutions of vCJD brain homogenate. Samples were incubated with the compound and 48 PMCA cycles (24 h) were done to asses prion replication by western blot. (**B**), The IC50 can be estimated from the experiment in panel A, by plotting the inhibitory activity (expressed as a last dilution in which signal is observed) versus the logarithm of the compound concentration. Negative control (Neg) consists of samples without addition of PrP^Sc^. Samples were subjected to one round of 48 PMCA cycles (24 h) and analyzed by western blot after PK digestion. NBH correspond to transgenic mice normal brain homogenate, used as a migration control. Lanes on the left of each blot represent molecular weight standards. Please see the original Western blot image in the Supplementary Materials.

Using PMCA, we can also estimate the half-maximal inhibitory concentration (IC50). For this purpose, we tested the inhibitory activity in prion replication of different dilutions of vCJD brain homogenate in the presence of distinct concentrations of MB (Figure 3A). The data shows that high concentrations of MB (>25 µM) completely block prion replication even when using high amounts of vCJD PrP^Sc^ (low dilutions of brain homogenate), whereas low MB concentrations (<2 µM) produced no significant effect on prion replication (Figure 3A). To calculate IC50 we plotted the inhibitory activity (expressed as the last dilution in which the signal is observed) versus the logarithm of MB concentration (Figure 3B). For MB, we estimated an IC50 of 7.7 µM against vCJD prions.

### 3.3. Initial Structure-Activity Relationship Studies

**Figure 4:**
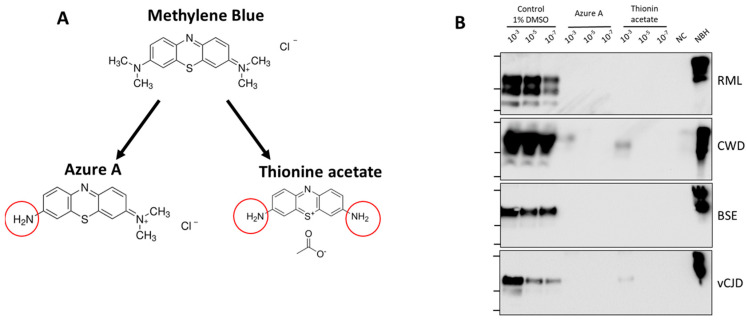
**Preliminary structure-activity relationship studies.** (**A**), Two close derivatives of MB, including azure A and thionine acetate were used to study the effect of chemical groups substitutions on MB activity. (**B**), Activity of the MB’s derivatives was studied as in Figure 2. Negative control (NC) consists of samples without addition of PrP^Sc^. Samples were subjected to one round of 48 PMCA cycles (24 h) and analyzed by western blot after PK digestion. NBH correspond to transgenic mice normal brain homogenate, used as a migration control. Lanes on the left of each blot represent molecular weight standards (34 KDa, 26 KDa and 17 KDa). Please see the original Western blot image in the Supplementary Materials.

To begin structure-activity relationship studies of the best inhibitor in this set, we searched for chemical derivatives of MB (3,7-bis(dimethylamino)-phenothiazin-5-ium chloride) and identified two compounds: azure A (N′,N′-dimethylphenothiazin-5-ium-3,7-diamine chloride) and thionine acetate (3,7-Diamino-5-phenothiazinium acetate) (Figure 4A) with similar chemical structure. We tested the effect of these derivatives in inhibiting prion replication of RML, CWD, BSE and vCJD PrP^Sc^. The results show that while azure A retains activity, thionine acetate has a lower activity for CWD and vCJD (Figure 4B). Interestingly, all three compounds completely inhibited RML and BSE prions at a concentration of 100 μM.

## 4. Discussion

PrDs remain 100% fatal and, hence, one of the largest unmet needs in the field is to identify drugs capable of stopping or slowing down the progression of these devastating diseases. The main obstacles to the development of effective anti-prion drugs are the rapidly progressing nature of the disease, the unorthodox nature of the prion infectious agent, the exponential increase in the quantity of prions during the disease because of self-propagating prion replication, the need for compounds to effectively cross the blood-brain barrier, and the rare and heterogeneous clinical presentation of the disease [6,7,43,44]. The key hallmark of PrDs is the misfolding and aggregation of the prion protein (PrP^Sc^), which can self-propagate its misfolding at the expense of the cellular prion protein (PrP^C^). Many studies have been conducted to identify and evaluate potential drug candidates [6,7,43,44]. A diversity of compounds and strategies have been studied, including various chemical and natural compounds targeting either PrP^C^,PrP^Sc^ or other putative players in the pathogenic mechanism [7,44,45,46]. Other proposed anti-prion treatments include passive and active immunization strategies, peptides, aptamers, and PrP^C^-directed RNA interference techniques. A recent article by Zattoni and Legname [7], described a complete overview of the different reported strategies, including the list of compounds and the patents filed. Despite many publications and patents, only 6 clinical trials have been conducted so far to assess the therapeutic utility of diverse compounds, including flupirtine, quinacrine, doxycycline, pentosane polysulfate, the prion protein monoclonal antibody PRN100 and an anti-sense oligonucleotide [43]. Unfortunately, none of these trials produced significant therapeutic benefits.

In this study, we tested the effect of eight chemical compounds that have been shown previously to alter prion replication in different model systems. Prion replication was measured by PMCA, using simultaneously prions from four different species: mouse RML, human vCJD, cattle BSE and cervid CWD. Our results showed that five out of eight compounds tested (Congo red, tannic acid, curcumin, quinacrine and MB) produced some anti-prion activity (Figure 2). Interestingly, some of these molecules inhibited differentially some prions and not others. Of note, four of the five compounds only inhibited replication of experimental RML prions. These results suggest that searching for molecules using exclusively experimental prions in rodent models may not be translatable to human prions. Out of the compounds tested, only MB produced a complete inhibition at the concentration tested in all prion species (Figure 2). However, since we measured prion replication at 24 h, it is not possible to rule out whether prion replication would still occur at longer times of the PMCA reaction. To study in more detail the inhibitory activity of MB, we used different concentrations of the molecule against vCJD prion replication (Figure 3). The results indicated that while concentrations of MB higher than 25 µM completely block prion replication, MB concentrations lower than 2 µM produced no significant effect on prion replication. We estimated the IC50 for MB as 7.7 μM (Figure 3). Finally, to begin attempting to understand the key chemical groups for activity, we tested the effect of two close derivatives of MB, azure A and thionine acetate (Figure 4). Thionine acetate only partially inhibited vCJD and CWD, while showing complete inhibition of RML and BSE prions (Figure 4). On the other hand, azure A showed an activity comparable to MB, except perhaps lower inhibitory capacity for CWD prions.

The results of this study suggest that PMCA is a powerful tool to identify and evaluate anti-prion compounds. The main advantage of PMCA is that it reproduces faithfully the process of prion replication while conserving the infectious and strain properties of prions. Furthermore, PMCA enables testing simultaneously various species and strains of prions, which is important since several molecules that were effective in murine prion models showed no activity in human studies. The main limitation of PMCA for drug screening is the low throughput of the assay. However, we believe this could be overcome by changing the format of the assay to ELISA plates, utilizing an easier readout (e.g., ELISA) and/or robotizing the assay. Another limitation is that PMCA can only test for inhibitors of prion replication and will miss molecules acting at other targets, such as prion neurotoxicity, clearance or expression.

## Data Availability

All data and materials generated in this study will be made fully available to the scientific community by contacting the corresponding author.

## Supplementary Materials

The following supporting information can be downloaded at: https://www.mdpi.com/article/10.3390/biom14091113/s1, The original Western blot image western blot image.
