# Supplementary material for: Screening of Anti-Prion Compounds Using the Protein Misfolding Cyclic Amplification Technology
Source: Biomolecules. 2024 Sep 4;14(9):1113. doi: 10.3390/biom14091113 (PMC11430292; doi:10.3390/biom14091113)
Supplement: Supplementary file 1 [file biomolecules-14-01113-s001.zip › biomolecules-3018157-supplementary.pdf]

Figure S1

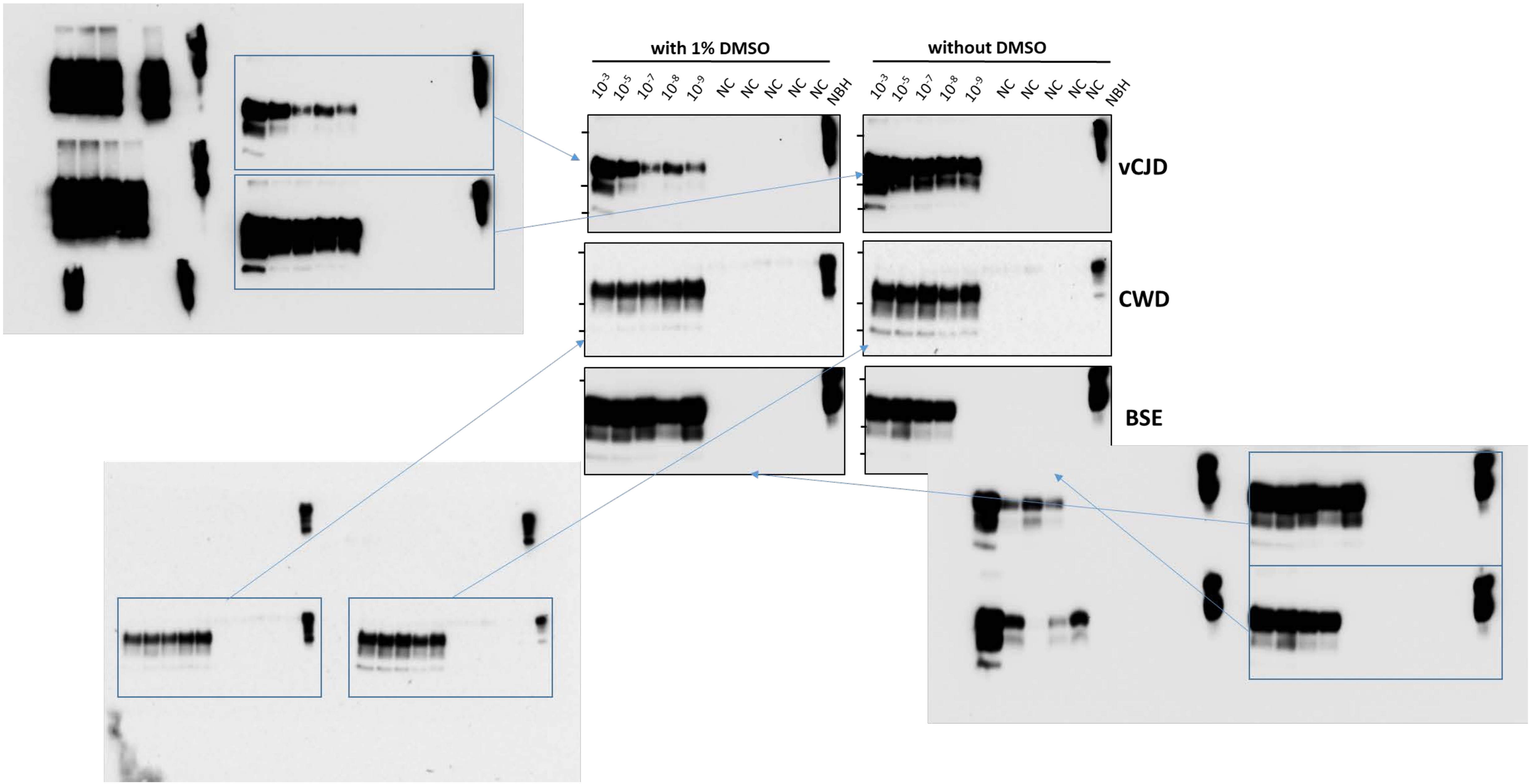
with 1% DMSO
without DMSO
10-3 10-5 10-7 10-8 10-9 NC NC NC NC NC NBH
10-3 10-5 10-7 10-8 10-9 NC NC NC NC NC NBH
vCJD
CWD
BSE

Figure S2. RML blots

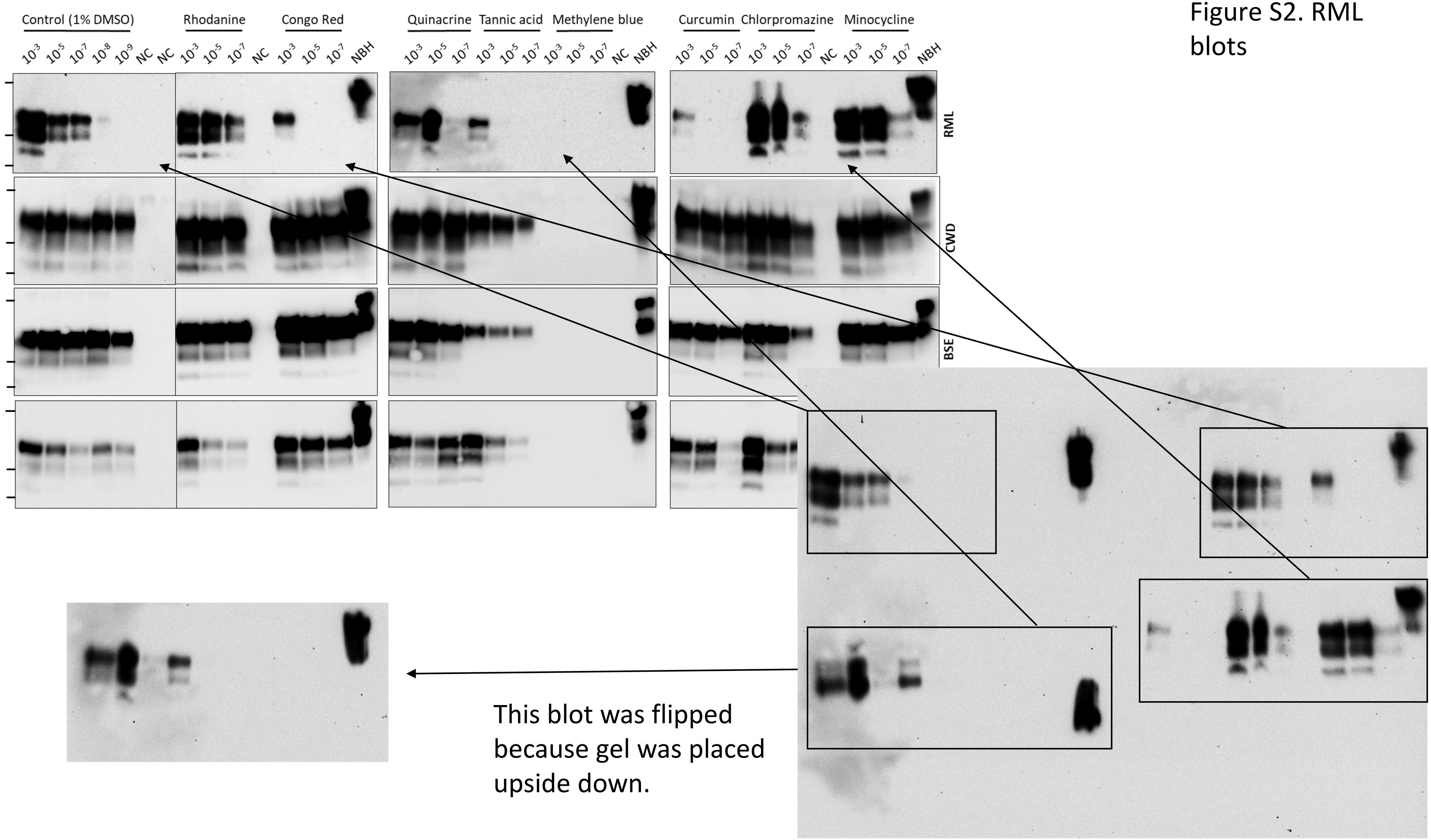

Figure S2. CWD blots

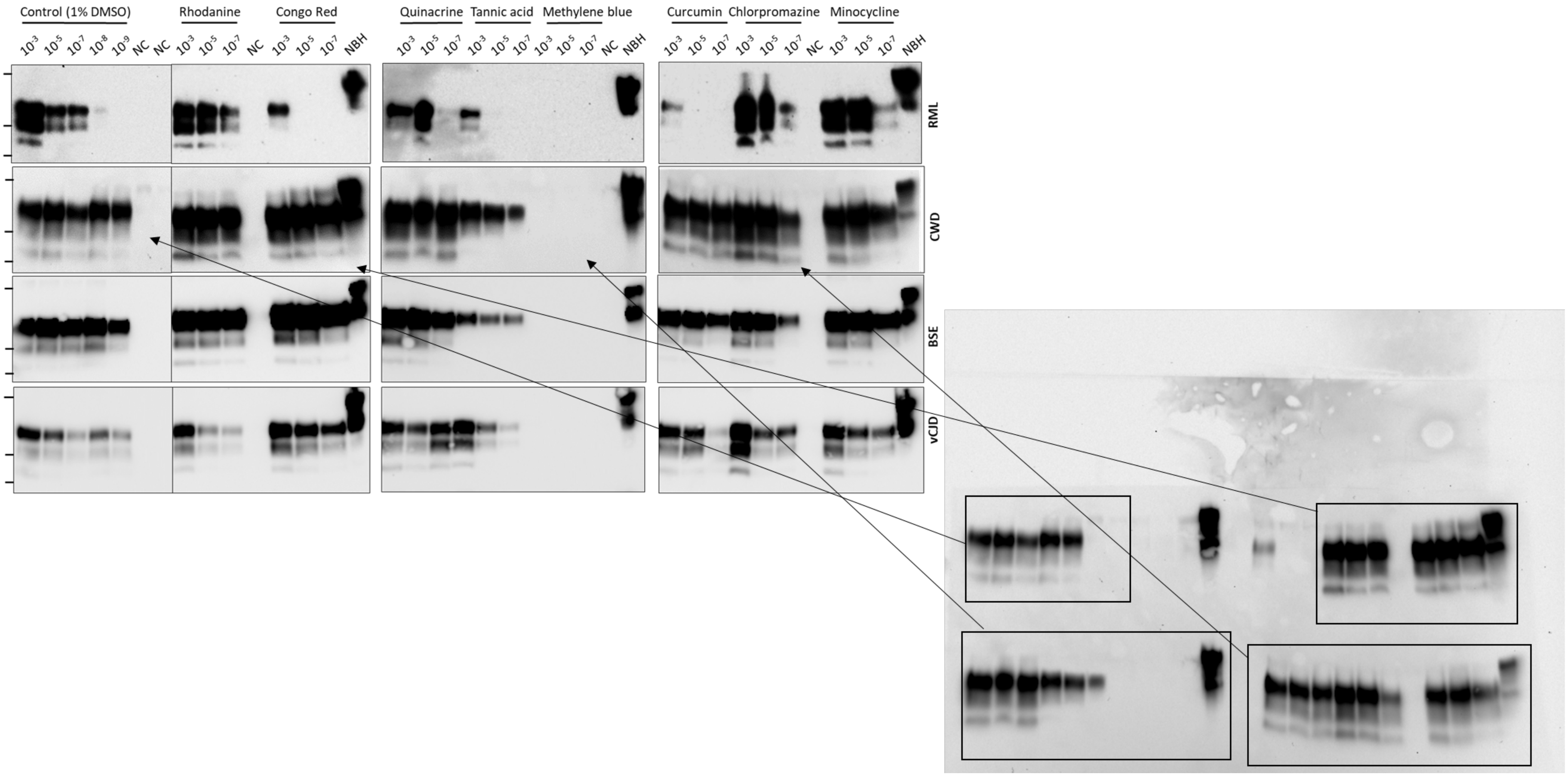

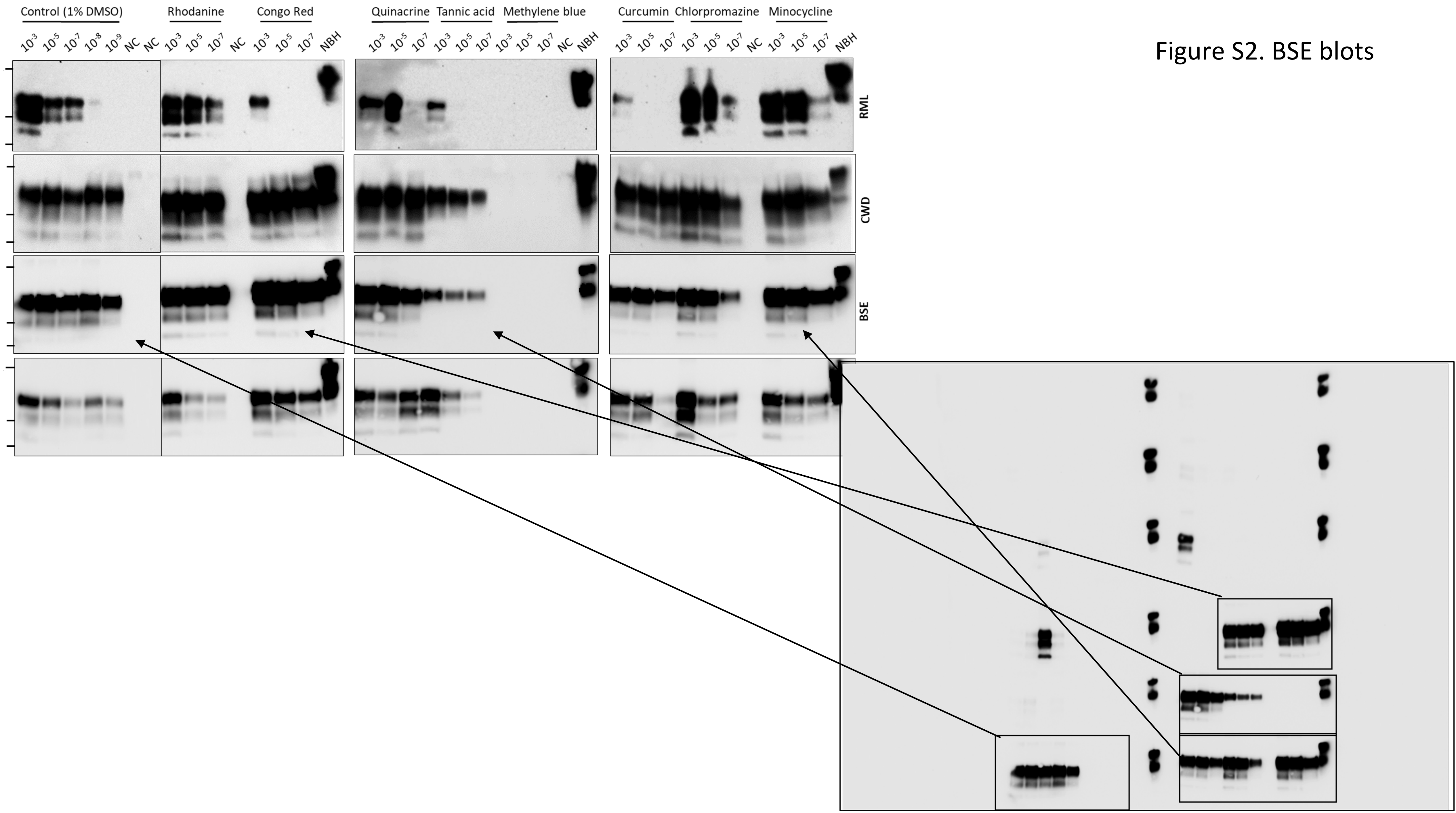

Figure S2. BSE blots

Figure S2. vCJD blots

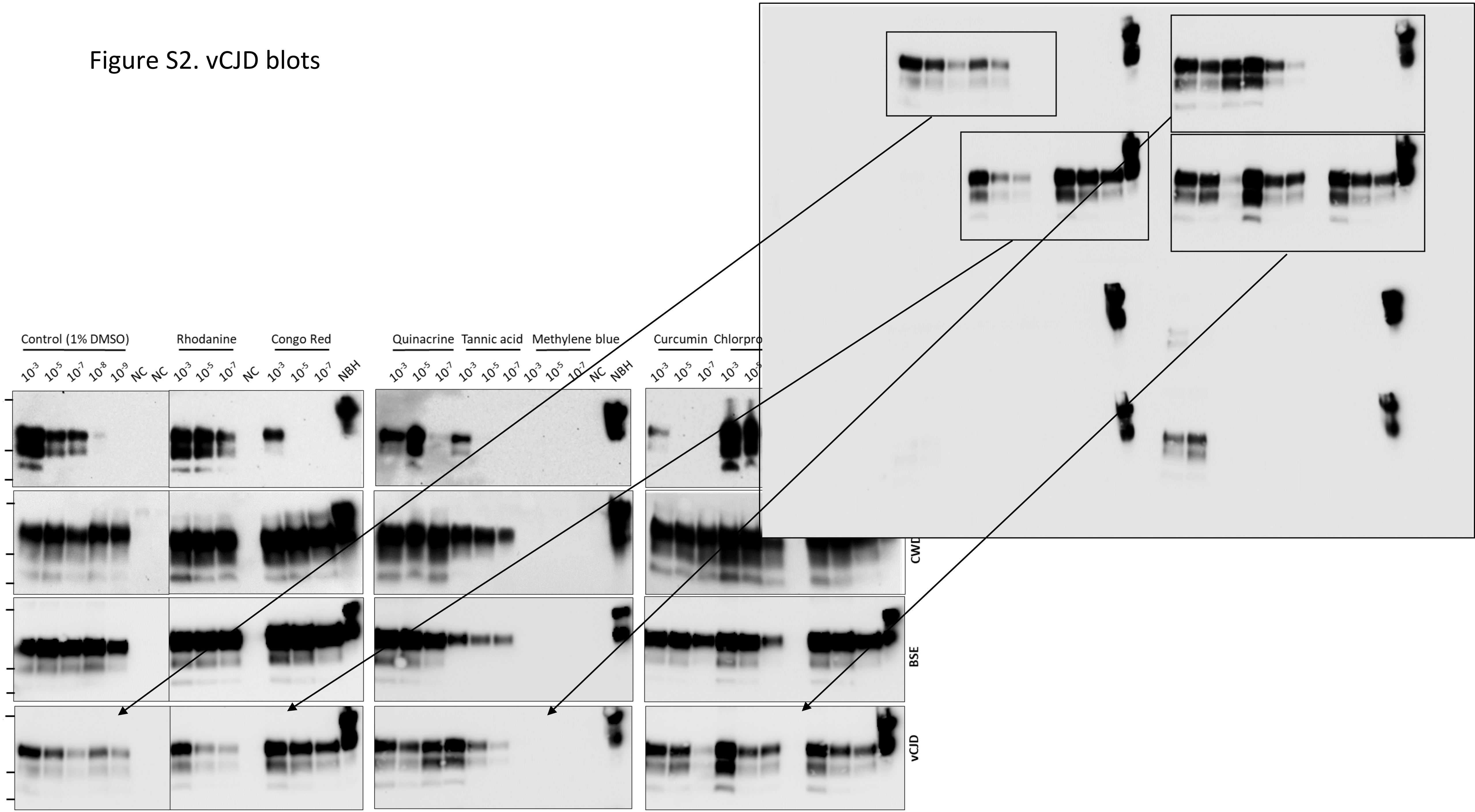

Figure S3

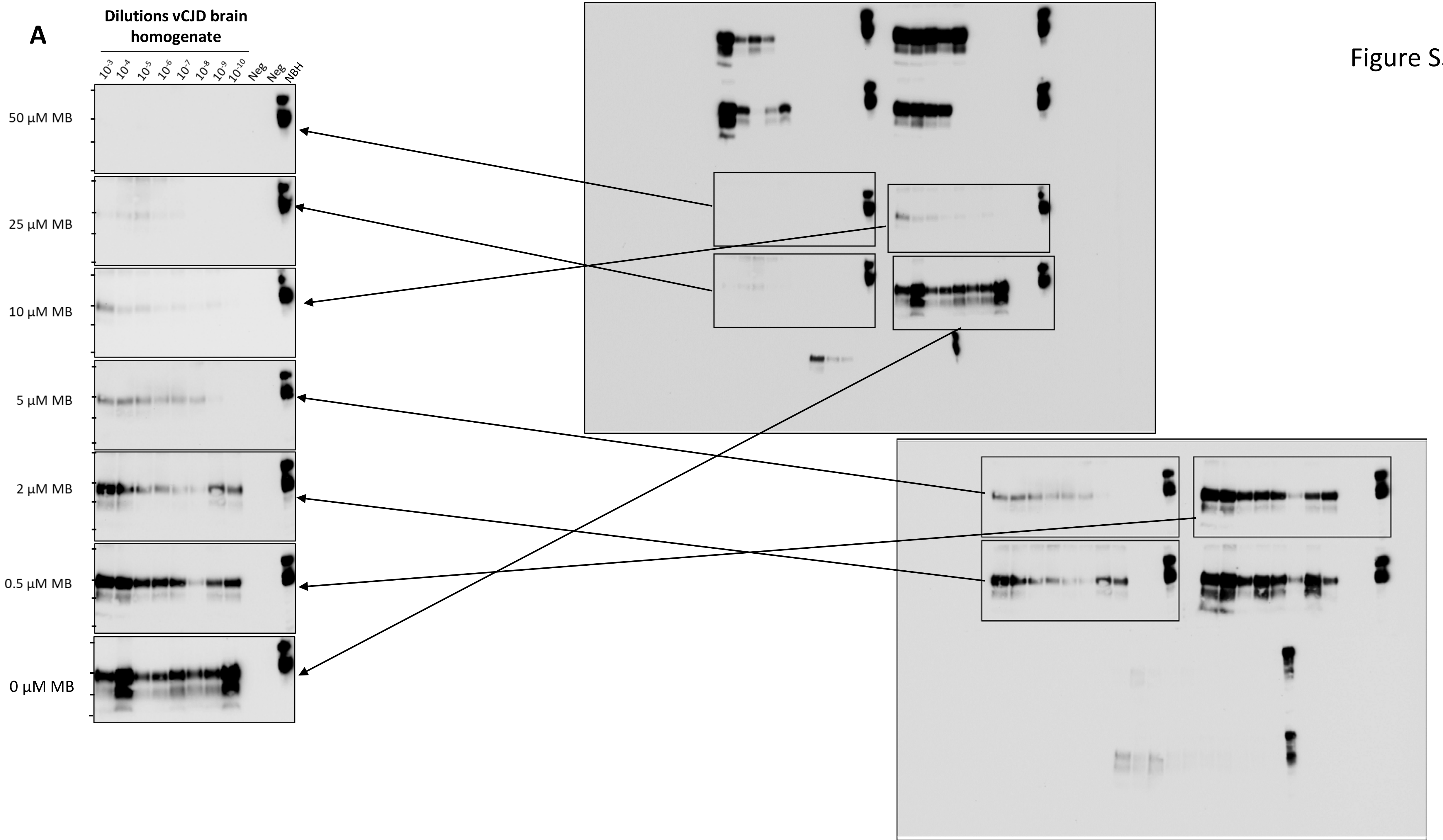
A
Dilutions vCJD brain homogenate
10^{-3} 10^{-4} 10^{-5} 10^{-6} 10^{-7} 10^{-8} 10^{-9} 10^{-10} Neg Neg NBH
50 µM MB
25 µM MB
10 µM MB
5 µM MB
2 µM MB
0.5 µM MB
0 µM MB

B

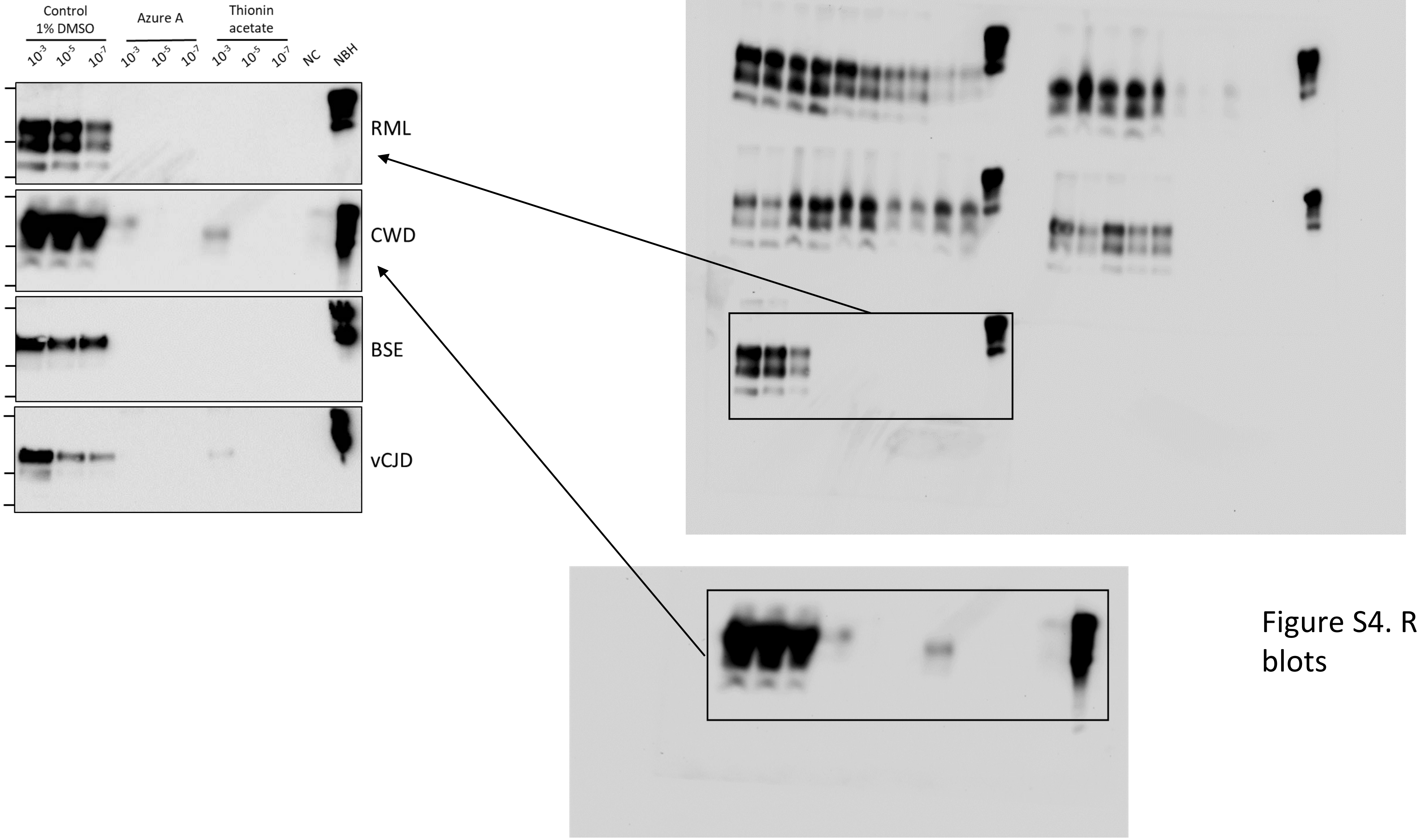

Figure S4. RML and CWD blots

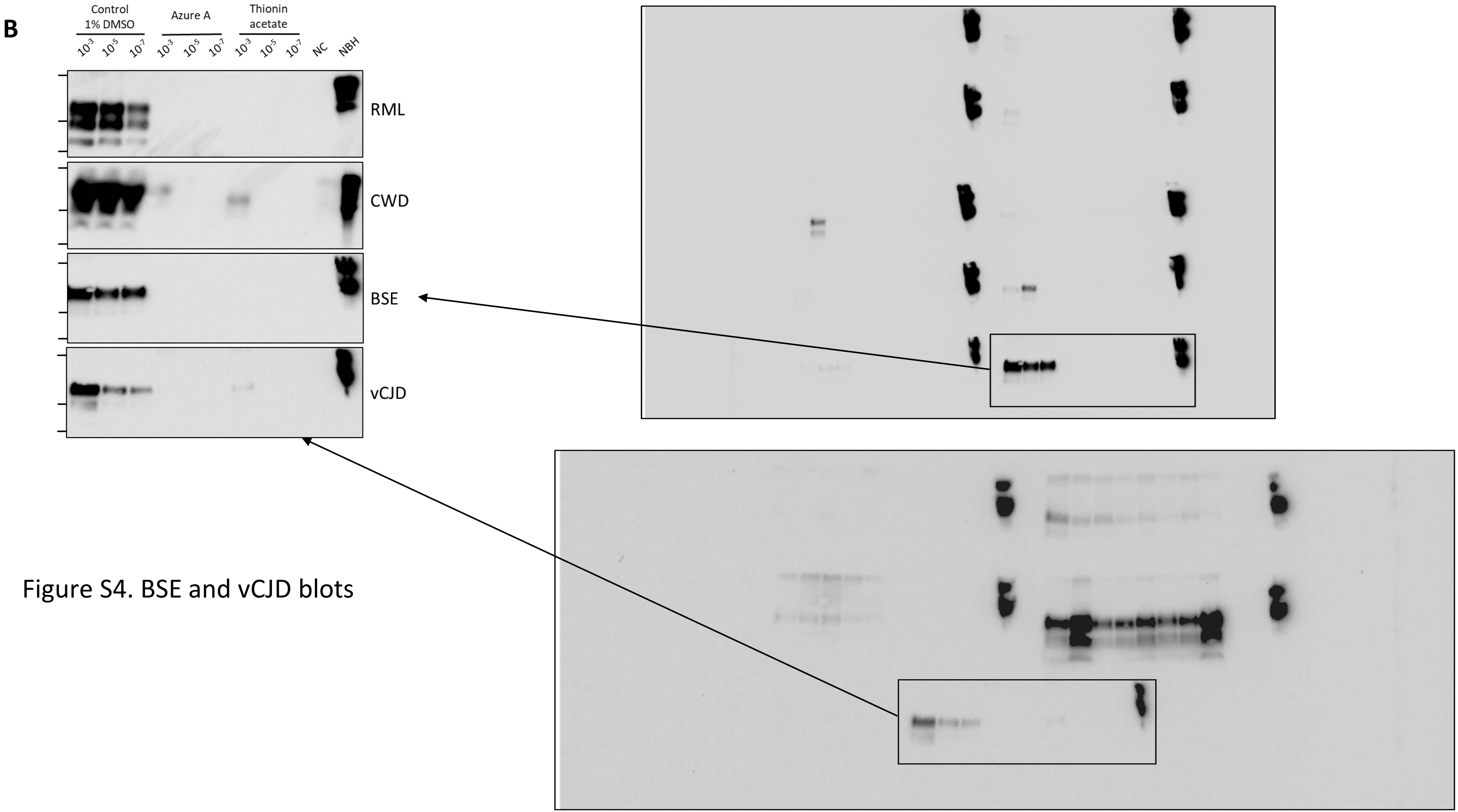

Figure S4. BSE and vCJD blots
